# Aerobic Training Modulates the Increase in Plasma Concentrations of Cytokines in response to a Session of Exercise

**DOI:** 10.1155/2021/1304139

**Published:** 2021-01-16

**Authors:** Tatiana Ramos Fonseca, Thiago Teixeira Mendes, Guilherme Passos Ramos, Christian Emmanuel Torres Cabido, Rodrigo Figueiredo Morandi, Fernanda Oliveira Ferraz, Aline Silva Miranda, Vanessa Amaral Mendonça, Antônio Lúcio Teixeira, Emerson Silami-Garcia, Albená Nunes-Silva, Mauro Martins Teixeira

**Affiliations:** ^1^Laboratório de Fisiologia do Exercício, Departamento de Educação Física (LAFISE/EEFFTO/UFMG), Belo Horizonte, Brazil; ^2^Laboratório de Imunofarmacologia, Departamento de Bioquímica e Imunologia (ICB/UFMG), Belo Horizonte, Brazil; ^3^Departamento de Educação Física (UFMA), São Luís, Brazil; ^4^Laboratório Interdisciplinar de Investigação Médica, Faculdade de Medicina (UFMG), Belo Horizonte, Brazil; ^5^Laboratório de Inflamação e Metabolismo (LIM/UFVJM), Teófilo Otoni, Brazil; ^6^Laboratório de Resolução da Resposta Inflamatória, Departamento de Morfologia (ICB/UFMG), Belo Horizonte, Brazil; ^7^Laboratório de Inflamação e Imunologia do Exercício (LABIIEx), Escola de Educação Física da Universidade Federal de Ouro Preto (UFOP), Ouro Preto, Brazil

## Abstract

Acute physical exercise can modulate immune function. For example, acute exercise is known to increase the circulating concentration of cytokines. Exercise is also known to modulate immune function chronically. It is not known whether exercise training can result in training of the immune system. Here, we investigated the effects of six weeks of aerobic training on cytokine responses induced by acute exercise until fatigue. Twelve healthy men performed a fatiguing exercise at the anaerobic threshold (AT) intensity. After the training period, the participants performed another bout of acute exercise at the same duration and intensity of the pretraining situation. The analysis was made at the beginning, end, and at 10, 30, and 60 minutes during the recovery period. Training at AT induced a gain of 11.2% of exercise capacity. Before training, a single bout of acute exercise induced a significant increase in plasma levels of cytokines, including IL-6, TNF-*α*, sTNFR1, IL-10, CXCL10, BDNF, leptin, resistin, and adiponectin. After six weeks of aerobic training, levels of IL-6, sTNFR1, BDNF, and leptin increased to a lesser extent after an acute bout exercise at the same absolute intensity as the pretraining period. Responses to the same relative exercise intensity were similar to those observed before exercise. These results show that aerobic training is associated with training of acute immune responses to acute exercise until fatigue.

## 1. Introduction

Regular physical exercise is thought to have important beneficial effects on human health. Indeed, over the past three decades, a significant body of evidence has demonstrated that regular physical exercise induces considerable changes in the health of the population [1–3]. In addition, the use of exercise has become a staple in the prevention and treatment options for delaying the development of health issues, such as overweight and obesity [3]. Studies have also shown that there is a strong association between the increase in physical inactivity and the emergence of modern chronic diseases in 20th century industrialized societies [1], and lack of exercise is considered a major risk factor for chronic diseases [2]. Many aspects of human health can be affected by exercise, including cardiovascular risk reduction [4], insulin sensitivity [5] and blood glucose homeostasis [6], weight loss and maintenance [7], and systemic inflammation [8–12]. In addition, physical exercise improves memory acquisition [13], regulates mood and anxiety, works as a strategy for reducing depression, enhances well-being [14], increases life longevity [15], improves learning [16], and improves cardiorespiratory fitness and biomarkers of cardiometabolic health [17].

Many studies have now clearly shown that many types of acute physical exercise induce remarkable alterations in the behavior of the immune system [18–25]. Acute physical exercise modifies the number and function of the circulating leukocyte pool [26, 27]. In addition, it has been shown that plasma level of many cytokines increases during and after a single acute exercise session [28–32]. The increase in the level of gene expression and local and circulating protein content is associated with the type and intensity of exercise and the health status of participants [33]. Different exercise protocols and load induce distinct responses in the concentration of cytokines in plasma [34–37].

Therefore, there is a large body of convincing evidence showing an interaction between physical exercise and the immune system (as seen by the systemic elevation of cytokines) during and after an acute physical exercise session [38–41]. This cytokine response is believed to be necessary for the formation of new vessels and the remodeling of skeletal muscle [42] and adipose tissue [43].

Chronic physical training is known to be associated with a gain of exercise capacity. However, it is not known whether physical training will impact on acute cytokine responses to an acute bout of exercise. The hypothesis of this study is that the acute increase in plasma levels of cytokines in response to an acute bout intense exercise is modulated by physical training. Fundamentally, this study aims to contribute to the concept that it is possible to train the innate immune system [44] and that exercise is an important physiological manner to do so.

Thus, the purpose of this study was to investigate the effects of physical exercise training on the modulation of cytokine responses to an acute bout of exercise. Indeed, it was our objective to ascertain whether exercise training would induce not only physical training, but also the “training” of the immune system, as assessed by measuring levels of cytokines in response to acute exercise stimulation.

To this end, we evaluated whether six weeks of aerobic training program would modify the cytokine (IL-6, TNF-*α*, sTNFR1, IL-10, CXCL10, BDNF, leptin, resistin, and adiponectin) responses induced by an acute bout of exercise performed until fatigue at anaerobic threshold intensity.

## 2. Materials and Methods

### 2.1. Participants and Study Design

Twelve young (22.5 ± 0.7 years) untrained males volunteered to participate, after recruitment through posters and social media at our university. Mean (±SEM) body mass, height, and VO_2PEAK_ were 71.9 ± 1.6 kg, 1.76 ± 0.02 m, and 45.5 ± 1.2 mL·kg^−1^·min^−1^, respectively. All participants were also asked to keep a food log of the 24 h preceding each acute exercise session. All procedures in this investigation were performed in accordance with the Declaration of Helsinki, Ethics Committee of Federal University of Minas Gerais approved all procedures (EC# 261/09), and all participants provided written informed consent after reading a detailed description of the protocol.

At pretraining, all participants were tested to determine VO_2PEAK_ and the anaerobic threshold (AT) (pretraining/intensity 1, I1) and performed acute exercise until fatigue at I1. There was a minimum of 48 h of rest between all exercise sessions and 72 h before the test until fatigue. Following six-week aerobic training program, all participants were subjected to retesting to define the posttraining VO_2PEAK_ and AT (posttraining/intensity 2, I2). After a minimum of 72 h for last training session, all participants performed two sessions of acute exercise (in different days and intensities): the first session had similar duration and absolute intensity (*w*) of initial test (pretraining) at I1 (to develop a similar total work when compared to the pretraining situation), and the second session was performed at I2 until fatigue (experimental design figure) (to develop a similar relative intensity at AT, due to training adaptations). All tests were always performed at the same time of the day (±1 h) to avoid circadian effects and in a room with controlled temperature and humidity (21–24°C, 50–70% RH).

Tests for physical characterization and tests until fatigue (time to fatigue) at anaerobic threshold (AT) before (AT-I1) and after (AT-I2) a six-week aerobic training. VO_2PEAK_: maximal oxygen peak; AT: anaerobic threshold, AT-I1: intensity of AT before training; AT-I2: intensity of AT posttraining. *Note. Posttraining* participants performed two tests until fatigue at different intensities: at AT-I2 and at AT-I1 (with similar duration on pretraining).

The participants performed 6 weeks of aerobic training, three times per week, at the I1 (about 75% VO_2PEAK_). The duration of the first training session was 24 min, and it increased to 39 min over the 6-week period (increase of 3 min per week) (Table 1). The training protocol was adapted from the study by Philp et al. [45], and the six-week period seems to be sufficient to induce significant adaptations [45, 46] in untrained participants. Each training session took place under the supervision of a researcher. All tests and aerobic training were performed on a cycle ergometer (Monark Ergomedic E-824E) previously calibrated, according to the manufacturer specifications, before each test and training.

Body mass and height were measured with a digital scale and a stadiometer (Filizola®). The body composition was estimated by the seven-skinfold method according to Jackson and Pollock [47]. The same researcher always performed the skinfold evaluation with a skinfold caliper (Lange®).

The participants were asked to drink 500 mL of water two hours prior to the beginning of all situations (experimental situations and training sessions) [48]. In all experimental situations, all participants were instructed to not drink any alcohol or beverages containing caffeine or perform any acute physical activity for at least 24 hours prior to the experiment's situation, to maintain the same diet in the evenings and mornings of the experimental situation, and each participant was asked about the experimental recommendations for food, alcohol, and caffeine consumption before starting experimental procedures. They were allowed to ingest water ad libitum in all experimental situations and training sessions, and before experimental situations, all participants were euhydrated (urine specific gravity ≤ 1020).

### 2.2. Assessment of VO_2PEAK_

Progressive exercise to fatigue, according to Balke and Ware [49], was performed to determine the VO_2PEAK_ before and after the aerobic training. The power started at 50 W and the workload was increased by 25 W·2 min^−1^. At the end of each stage, the rate of perceived exertion (RPE) according to Borg [50] was evaluated. Before each test, the participants rested sitting on a chair (5 min) to set the equipment for O_2_ uptake analysis (K4b2; Cosmed®, Italy) and heart rate (HR) (Polar Team System, Finland). Ventilation variables were measured, breath-by-breath, and the gas analyzer was calibrated before each test. The highest VO_2_ of the exercise was considered the VO_2PEAK_.

At least two of the following criteria had to be met to determine the VO_2PEAK_: (1) no increase in VO_2_ or HR despite increased exercise intensity, (2) RPE greater than 17 on the 6–20 scale, and (3) respiratory exchange ratio greater than 1.10 [51].

### 2.3. Assessment of AT and Responses at Acute Exercise until Fatigue

The AT was determined as described in Mendes et al. [52] and was considered the exercise intensity of maximal lactate steady state (MLSS). The MLSS is considered for some authors the gold standard protocol to determine AT [53–55] and an intensity that could be maintained for about 45–60 min [56] that is probably able to induce immune response. All participants performed two to five 30 min exercise sessions at constant intensities to determine the AT with a minimum of 48 h between each test. During the AT tests, blood samples (30 *μ*L) were collected from the ear lobe for blood lactate concentration analysis prior to the beginning of the exercise and at 5 min intervals until the end of the test. The highest power output at which the blood lactate concentration increased to less than 1 mM during the last 20 min of exercise was defined as the MLSS/AT [53, 54]. The AT was determined with a precision of 15 W. Blood was stored at −20°C in tubes containing 60 *μ*L NaF (1%) and later analyzed in duplicate for lactate concentration using an electroenzymatic analyzer (YSI 1500 STAT, Yellow Springs, Ohio, USA).

The AT was evaluated before and after the training program (intensity I and II for pre- and posttraining, I1 and I2) and corresponded to ∼75% VO_2PEAK_. The AT was assessed to relativize the exercise intensity and to create an exercise until fatigue length of 30–60 minutes and similar times of exercise before and after training.

For the acute exercises (pretraining at I1, posttraining at I1, and posttraining at I2), fatigue was considered as an individual's inability to maintain pedal frequency of 60 rpm, RPE of 20, or the test interruption requested by the subject. Venous blood samples were collected before (0 min) and at the end of the exercise (End) and at 10, 30, and 60 minutes during the recovery period to determine plasma levels of cytokines, chemokines, and lactate. Participants were not allowed to know any physiological variables, nor their exercise time, throughout all tests.

### 2.4. Correction for Plasma Volume Shifts and ELISA

Peripheral venipuncture was performed with a catheter (Angiocath®, BD-Becton Dickinson, 22G, USA). The blood was then centrifuged, and the plasma was stored in Eppendorf tubes in a freezer at −20°C. Changes in plasma volume were calculated from measurements of hemoglobin and hematocrit according to the method described by Dill and Costill [57], and plasma levels of cytokines and chemokines measurements were corrected accordingly.

Plasma cytokines and chemokines were measured by ELISA according to the procedures supplied by the manufacturer (DuoSet, R & D Systems, Minneapolis, MN, USA). The results were presented in picograms per ml (pg/mL). IL-6, TNF-*α*, sTNFR1, IL-10, CXCL10/IP-10, BDNF, leptin, resistin, and adiponectin were measured in this study. Blood lactate was analyzed using an electroenzymatic analyzer (YSI 1500 STAT, Yellow Springs, Ohio, USA).

### 2.5. Statistical Analysis

Normality was assessed using the Ryan-Joiner test, homoscedasticity was assessed using Levene's test, and sphericity was examined using Mauchly's test prior to all analyses. Cytokines and chemokines data did not achieve normal distribution and were analyzed by nonparametric tests. The variable IL-6 had normal distribution after logarithmic transformation.

To analyze the effects of the training program in the VO_2PEAK_, body mass, and percentage of body fat (before and after training) paired *t*-test was used. Additionally, Cohen's *d* effect-size (ES) estimates were calculated to assess the magnitude of difference between the experimental conditions. ES was calculated through mean differences [58] and was considered trivial (ES < 0.2), small (ES: 0.2–0.6), medium (ES: 0.6–1.2), or large (ES ≥ 1.2) [59]. Additionally, Cohen's effect size (ES) and confidence intervals of ES (CI) of 95% were calculated to assess the magnitude of difference between the experimental conditions..

To compare exercise intensities and physiological responses for exercise sessions at pretraining at I1 and posttraining at I1 and I2, one-way ANOVA with repeated measures was used. To analyze IL-6 (log) responses, two-way ANOVA with repeated measures was used. A Student–Newman–Keuls post hoc analysis was used when necessary. To analyze other cytokines and chemokines (TNF-*α*, sTNFR1, IL-10, CXCL10, BDNF, leptin, resistin, and adiponectin), a nonparametric Friedman test was applied. To test the association between the variables, a Pearson product-moment correlation coefficient (parametric–log IL-6) was used.

The results of parametric analysis are presented as mean ± standard error of mean and nonparametric analysis of cytokines, and chemokines are presented as median and individual response. The significance level used was *p* < 0.05. Statistical analysis was performed in Sigma Plot v. 13 and SPSS v. 22.

## 3. Results

### 3.1. Aerobic Training

**Figure 1:**
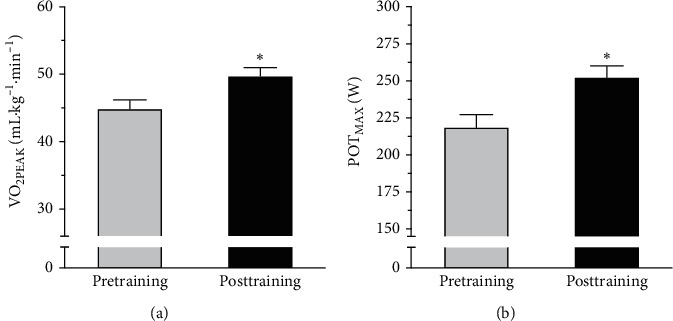
(a) Maximal oxygen uptake (VO_2PEAK_) and (b) maximal power output (POTMAX) before (pre) and after (post) aerobic training. ^*∗*^*p* < 0.01.

Twelve healthy participants performed six weeks of aerobic training on a cycle ergometer at their anaerobic threshold (AT) before the training session (referred to as I1). We compared anthropometric parameters before and after the period of aerobic training, and no differences were detected in body mass (71.9 ± 1.7 vs 71.4 ± 1.6 kg, ES = −0.30; IC = −1.55–0.94) and percentage of body fat (14.2 ± 1.5 vs 13.2 ± 1.3%, ES = −0.71; IC = −1.99–0.57). All the participants completed six weeks (18 sessions) of aerobic training, and this was associated with an increase of 11.2 ± 6.6% in VO_2PEAK_ (44.9 ± 4.8 vs. 49.8 ± 4.5; *p* < 0.05; ES = 1.06; IC = −0.27–2.38) (Figure 1(a)) and in maximal power (POTMAX) (218.7 ± 30.8 vs. 252.4 ± 27.9, *p* < 0.05; ES = 1.15; IC = −0.19–2.49) (Figure 1(b)), suggesting that aerobic training was effective in generating cardiovascular and pulmonary adaptations.

All participants were subjected to acute exercise (cycle ergometer), as a stressor response, before training (pretraining) and 6 weeks after exercise training at AT intensities. Physiological responses to exercise at I1 and posttraining I1 and I2 sessions are shown in Table 2. During the posttraining I1 session, participants performed at lower percentage of maximal oxygen uptake (% VO_2PEAK_) and maximal power (% POTMAX) when compared to the posttraining I2 session. In addition, there were lower values of RPE and HR (Table 2) in the posttraining session I1, demonstrating that the physiological stress appeared to be lower during the posttraining session I1. The latter findings are consistent with the gain in performance observed in Figure 1. In addition, the data suggest that the physiological stress was similar in pretraining at I1 and posttraining at I2 sessions.

**Figure 2:**
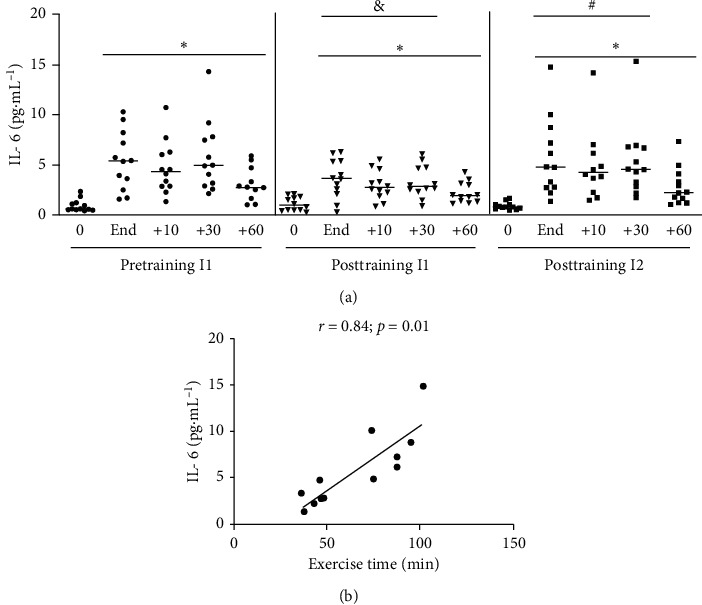
(a) IL-6 plasmatic concentration at pretraining and posttraining, before, after, 10, 30, and 60 min after acute exercise. Pretraining (physical exercise performed until fatigue at anaerobic threshold (I1) before 6 weeks of training); posttraining 1 (aerobic acute exercise performed at the same absolute intensity (I1) and duration of pretraining) and posttraining 2 (after aerobic acute exercise performed until fatigue at anaerobic threshold of posttraining (I2)). ^*∗*^*p* < 0.05 from resting period (0) for the same situation; ^&^*p* < 0.05 from pretraining for the same time point; ^#^*p* < 0.05 from posttraining I1 for the same time point. *n* = 12. (b) Pearson product-moment correlation between IL-6 and exercise time to fatigue. *n* = 12.

**Figure 3:**
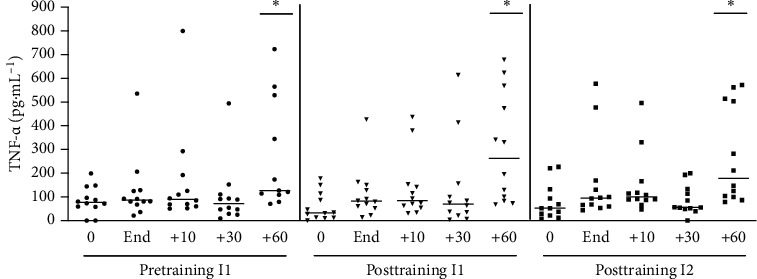
TNF-*α* plasmatic concentration at pretraining and posttraining, before, after, 10, 30, and 60 min after acute exercise. Pretraining (physical exercise performed until fatigue at anaerobic threshold (I1) before 6 weeks of training); posttraining 1 (aerobic acute exercise performed at the same absolute intensity (I1) and duration of pretraining) and posttraining 2 (after aerobic acute exercise performed until fatigue at anaerobic threshold of posttraining (I2)). ^*∗*^*p* < 0.05 from resting period (0) for the same situation. *n* = 12.

**Figure 4:**
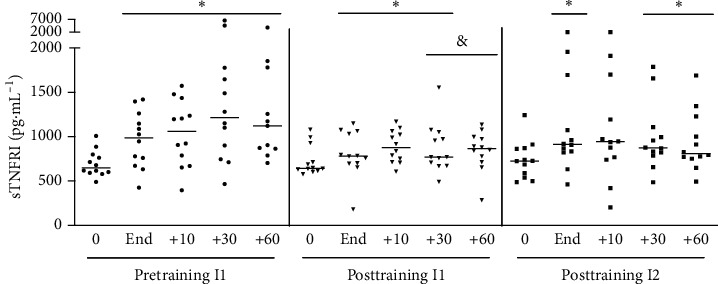
sTNFR1 plasmatic concentration at pretraining and posttraining, before, after, 10, 30, and 60 min after acute exercise. Pretraining (physical exercise performed until fatigue at anaerobic threshold (I1) before 6 weeks of training); posttraining 1 (aerobic acute exercise performed at the same absolute intensity (I1) and duration of pretraining) and posttraining 2 (after aerobic acute exercise performed until fatigue at anaerobic threshold of posttraining (I2)). ^*∗*^*p* < 0.05 from resting period (0) for the same situation; ^&^*p* < 0.05 from pretraining for the same time point. *n* = 12.

**Figure 5:**
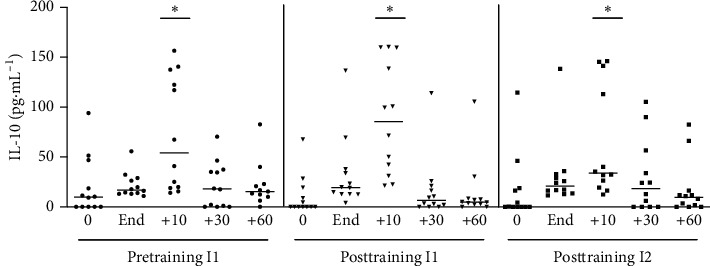
IL-10 plasmatic concentration at pretraining and posttraining, before, after, 10, 30, and 60 min after acute exercise. Pretraining (physical exercise performed until fatigue at anaerobic threshold (I1) before 6 weeks of training); posttraining 1 (aerobic acute exercise performed at the same absolute intensity (I1) and duration of pretraining) and posttraining 2 (after aerobic acute exercise performed until fatigue at anaerobic threshold of posttraining (I2)). ^*∗*^*p* < 0.05 from resting period (0) for the same situation. *n* = 12.

**Figure 6:**
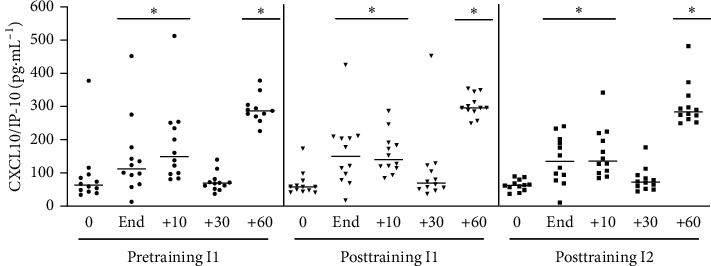
Plasma level of CXCL10/IP-10. Pretraining (physical exercise performed until fatigue at anaerobic threshold (I1) before 6 weeks of training); posttraining 1 (aerobic acute exercise performed at the same absolute intensity (I1) and duration of pretraining) and posttraining 2 (after aerobic acute exercise performed until fatigue at anaerobic threshold of posttraining (I2)). ^*∗*^*p* < 0.05 from resting period (0) for the same situation. *n* = 12.

**Figure 7:**
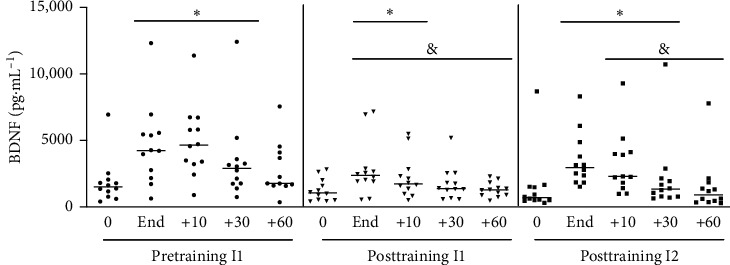
Plasma levels of BDNF. Pretraining (physical exercise performed until fatigue at anaerobic threshold (I1) before 6 weeks of training); posttraining 1 (aerobic acute exercise performed at the same absolute intensity (I1) and duration of pretraining) and posttraining 2 (after aerobic acute exercise performed until fatigue at anaerobic threshold of posttraining (I2)). ^*∗*^*p* < 0.05 from resting period (0) for the same situation; ^&^*p* < 0.05 from pretraining for the same time point; *n* = 12.

**Figure 8:**
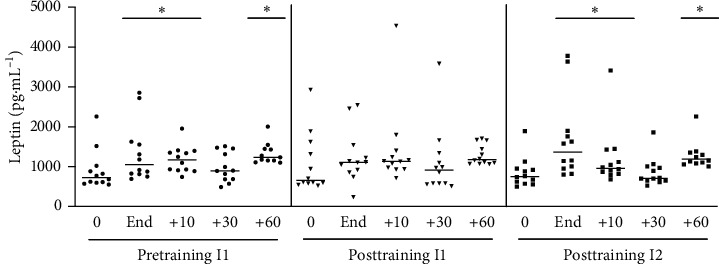
Leptin levels after acute physical exercise: the leptin levels were measured before and after exercise at times of 10, 30, and 60 minutes in postexercise recovery. Pretraining (physical exercise performed until fatigue at anaerobic threshold (I1) before 6 weeks of training); posttraining 1 (aerobic acute exercise performed at the same absolute intensity (I1) and duration of pretraining) and posttraining 2 (after aerobic acute exercise performed until fatigue at anaerobic threshold of posttraining (I2)). ^*∗*^*p* < 0.05 from resting period (0) for the same situation; *n* = 12.

**Figure 9:**
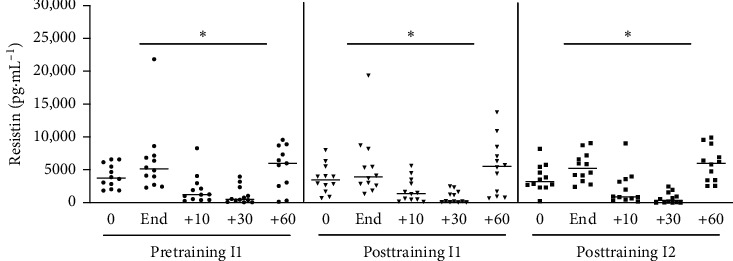
Resistin level after acute physical exercise: the resistin levels were measured before and after exercise at times of 10, 30, and 60 minutes in postexercise recovery. Pretraining (physical exercise performed until fatigue at anaerobic threshold (I1) before 6 weeks of training); posttraining 1 (aerobic acute exercise performed at the same absolute intensity (I1) and duration of pretraining) and posttraining 2 (after aerobic acute exercise performed until fatigue at anaerobic threshold of posttraining (I2)). ^*∗*^*p* < 0.05 from resting period (0) for the same situation; *n* = 12.

**Figure 10:**
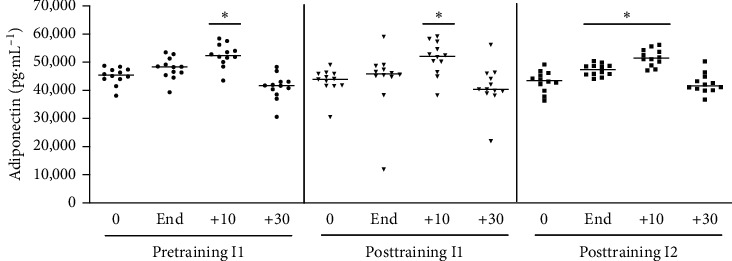
Plasma adiponectin levels after acute physical exercise: the plasma adiponectin levels were measured before and after exercise at times of 10, 30, and 60 minutes in postexercise recovery. Pretraining (physical exercise performed until fatigue at anaerobic threshold (I1) before 6 weeks of training); posttraining 1 (aerobic acute exercise performed at the same absolute intensity (I1) and duration of pretraining) and posttraining 2 (after aerobic acute exercise performed until fatigue at anaerobic threshold of posttraining (I2)). ^*∗*^*p* < 0.05 from resting period (0) for the same situation; *n* = 12.

In order to investigate the effects of aerobic training on blood circulating cytokines, we evaluated levels of cytokines previously known to elevate after acute exercise [60]. At rest, there were no detectable differences in blood concentration of the evaluated cytokines, IL-6, TNF-*α*, sTNFR1, IL-10, CXCL10, BDNF, leptin, resistin, and adiponectin, at pretraining, posttraining I1, and posttraining I2. Levels of cytokines increased significantly at the end of an acute bout of exercise or immediately thereafter (Figures 2–10). As shown in greater detail below, it is important to notice the different behavior of certain cytokines when comparing the response to an acute bout of exercise before and after a period of training.

#### 3.1.1. IL-6

IL-6 peaked at the very end and remained high until 60 min after the end of acute exercise (Figure 2). Levels of IL-6 at the end of exercise had a positive correlation with duration of the exercise (*r* = 0.88; *p*=0.01; ES = 3.71) (data not shown). After the training period, all individuals were subjected to acute exercise to the same intensity as before training (posttraining-I1). As seen in Figure 2, the concentration of IL-6 increased after exercise, but the median increase was significantly lower and dropped considerably faster when compared to the pretraining situation. When the individuals were exposed to the new level of I2 (posttraining I2), the profile and median concentration of the IL-6 response were similar to those observed in the pretraining situation at I1 (Figure 2). Levels of IL-6 at the end of exercise at post-I2 had a positive correlation with duration of the exercise (*r* = 0.88; *p*=0.01; ES = 3.71) (Figure 3)

#### 3.1.2. TNF-*α*

There was an increase of TNF-*α* levels only at 60 min after the end of exercise, and the intensity of increase was similar in all three groups (Figure 3); i.e., the level of TNF-*α* increased only 60 minutes after exercise, and median elevation was similar in the situation before and after training. There were no changes in IL-1*β* induced by acute exercise under our experimental conditions (data not shown).

#### 3.1.3. sTNFR1

Plasma concentrations of sTNFR1 elevated immediately after exercise and remained high at 10, 30, and 60 minutes after the finish of acute exercise (Figure 3). After six weeks of aerobic training, level of sTNFR1 also increased in the posttraining I1 situation but returned to the basal level faster than the pretraining situation (Figure 4). When the participants were exposed to the new level of I2 (posttraining I2), the profile and concentration of the sTNFRq response were similar to those observed in the pretraining situation at I1 (Figure 2).

#### 3.1.4. IL-10

IL-10 was only elevated 10 minutes after acute exercise, and the size of the response was similar in all three situations, i.e., before and after training and at the two levels of intensity (Figure 5).

#### 3.1.5. CXCL10/IP-10

We evaluated the effects of acute exercise on the levels of the three chemokines: CXCL8/IL-8, CCL2/MCP-1, and CXCL10/IP-10. Acute exercise did not significantly affect the circulating levels of CXCL8/IL-8 and CCL2/MCP-1 under our experimental conditions (data not shown). Therefore, these chemokines were not evaluated after aerobic training. However, acute exercise induced elevation of the levels of CXCL10/IP-10 in plasma in an interesting bimodal manner. The level of IL-10 first peaked at 10 minutes after exercise; then, it returned to baseline, and another peak of greater intensity was observed at 60 minutes after exercise (Figure 6). Overall, the response was similar in the three groups; i.e., exercise-induced elevations of CXCL10 were not altered by training.

#### 3.1.6. BDNF

As seen in Figure 7, resting (0 min) levels of BDNF were similar in all sessions, suggesting that baseline levels of this cytokine were not altered by aerobic training. In response to acute exercise, plasma levels of BDNF increased significantly and peaked at the end of exercise and at 10 minutes of the recovery period. Overall, levels of BDNF at the peak were twice as high as those at baseline. Thereafter, levels of BDNF fell and reached baseline levels again at 60 minutes after recovery. In the posttraining I1 session, in which individuals were subjected to equal absolute potency but a decreased stressor stimulus, levels of BDNF still increased at the end of exercise and at 10 minutes of recovery. However, the increase was significantly lower than that observed in the pretraining session (Figure 7).

In the posttraining I2 session, in which individuals were subjected to a similar relative potency and to a similar stressor stimulus, levels of BDNF curve peaked at the end of exercise, and this was similar to that of the pretraining session. However, levels of BDNF dropped rapidly and were significantly different from those found in the pretraining session at all times of recovery.

#### 3.1.7. Leptin

Acute physical exercise until fatigue was accompanied by an increase in plasma concentrations of leptin that peaked at the end of physical exercise and then again at 60 minutes during postexercise recovery (Figure 8). In the posttraining period, there was no significant increase in the concentration of leptin when these individuals were exposed to the same absolute intensity of the pretraining period (posttraining I1) (Figure 8). On the other hand, exposing participants to the same relative intensity of the pretraining until fatigue (posttraining I2) resulted in plasma concentrations of leptin, which were similar to those concentrations measured during acute exercise stimulation at the pretraining session.

#### 3.1.8. Resistin

Acute physical exercise was associated with increase of plasma resistin concentrations at the end of exercise and then at 90 minutes of postexercise recovery period (Figure 9). The same kinetics and concentration of resistin were observed after training when individuals were exposed to the same absolute or relative intensity of acute exercise of the pretraining period.

#### 3.1.9. Adiponectin

Adiponectin concentration increased after acute physical exercise, only 10 minutes after exercise, in participants with no training (Figure 10). A similar profile was observed after 6 weeks of aerobic training with the same absolute or relative intensity of pretraining acute exercise stimulation.

## 4. Discussion

The major findings of the current study can be summarized as follows: (i) A six-week period of aerobic training at AT (around 75% VO_2PEAK_) was able to induce performance improvement. (ii) Acute exercise at AT until fatigue induced a significant increase in circulating concentration of the cytokines IL-6, TNF-*α*, sTNFR1, IL-10, CXCL10, BDNF, leptin, resistin, and adiponectin in plasma, albeit with different kinetics of increase. (iii) For the first time, we were able to show that six weeks of moderate aerobic exercise training at AT was able to modulate acute exercise-induced elevation of certain cytokines in such way that the elevation of plasma levels of certain cytokines (IL-6, sTNFR1, BDNF, and leptin) was altered by aerobic training, but not others (TNF-*α*, IL-10, CXCL10, resistin, and adiponectin).

In this study, six weeks of aerobic training performed at 75% of VO_2PEAK_ induced a significative improvement of 11.2 ± 6.6% and a medium ES (ES = 1.06) of exercise capacity, as assessed by maximal oxygen consumption. All participants showed an improvement of maximal oxygen consumption and power at AT (14.7 ± 8.9% improvement of initial AT) indicating the effects of aerobic training in AT and maximal oxygen consumption. This was a significant gain of exercise capacity in a short period of time and is comparable to other studies evaluating increase in performance [60]. Therefore, the findings of the current study may be extrapolated to other types of training with similar gain of performance.

In our study, acute exercise to fatigue was associated with an increase in circulating concentrations of several cytokines, including IL-6, TNF-*α*, sTNFR1, IL-10, CXCI10/IP-10, BDNF, leptin, adiponectin, and resistin. This elevation is similar to that found by other studies [28–30, 61–63] and is consistent with the idea that acute exercise may function as an acute inflammatory stimulus [64–66]. Elevated cytokines included proinflammatory molecules (IL-6, TNF-*α*), molecules with anti-inflammatory activity (IL-10, sTNFR1), adipokines (leptin, adiponectin, resistin), and neurotrophic factors (BDNF). A meta-analysis study showed that resting concentrations of peripheral blood BDNF were higher after training process. Subgroup analyses also suggested a significant effect in aerobic but not resistance training interventions. It appears that aerobic but not resistance training interventions increased resting BDNF concentrations in peripheral blood chronically [67].

In our study, the exercise session was able to elevate the levels of leptin. Physical exercise and/or training can reduce fat mass, play a significant role in energy expenditure, and affect hormonal concentrations (insulin, cortisol, growth hormone, catecholamines, testosterone, etc.) and metabolites (free fatty acids, lactic acid, triglycerides, etc.). For all these reasons we believe that physical exercise and training could modify the leptin response depending on several factors such as intensity and duration of exercise session [68].

Recently, an elegant paper using “omics” analysis investigated 2, 15, 30, and 60 minutes after the end of aerobic exercise and showed an acute elevation in some molecules [69].

As example of the cytokine responses to exercise, the concentration of IL-6 peaked at the end of the acute exercise protocol. Thereafter, the concentration of this cytokine was kept high until 30 minutes, with a drop at 60 minutes of the recovery period, although it was still higher than basal levels (see Figure 2). An increase of IL-6 concentration in plasma after exercise has been shown using different exercise protocols [70–72]. The increase of IL-6 in plasma appears to depend on exercise intensity and duration [32] and is consistent with the suggested role of this cytokine in mobilizing energy and muscle hypertrophy [63]. The role of other molecules in the context of exercise is less understood, but it is believed that this acute inflammatory response may be relevant for metabolic and adaptive responses of the muscle, liver, and adipose tissue [73].

There were some differences in the time point at which the elevation of the concentration of cytokines occurred, when compared with other authors [27, 31, 32]. These differences may be explained by the type and intensity of exercise, which will determine the intensity of the inflammatory stimulation. In this regard, it is clear that our acute exercise protocol was capable of inducing a robust inflammatory stimulus followed by systemic increase in concentration of certain cytokines.

In the posttraining protocol, cytokine responses induced by acute exercise followed two general patterns: (i) There were cytokines which were elevated in a similar manner before and after training, if the individuals were subjected to the same relative or absolute acute exercise, and these included TNF-*α*, IL-10, CXCL10, resistin, and adiponectin. (ii) There were cytokines which responded in a different manner, if the posttraining were performed at the same absolute level of intensity of acute exercise as that given before the training; these included IL-6, sTNFR1, and leptin. In these individuals, the elevation of concentration of cytokines was smaller, or the return to baseline was faster. In contrast, they still had a strong response that was similar to that of the pretraining period, if they were again exposed to the same relative intensity level; i.e., there was training for cytokine responses as there was training for exercise.

Evaluation of these data clearly show that physical training induced no absolute and straightforward changes in cytokine responses to acute inflammatory stimulation (acute exercise); i.e., there were no major changes in proinflammatory vs anti-inflammatory molecules, or molecules that most likely induce metabolic changes versus those that do not. Indeed, the molecules that undergo training in their response are rather diverse in their nature and molecular actions. IL-6 is clearly active on the muscle and metabolic tissues [62], whereas sTNFR1 would tend to counteract these effects by decreasing TNF-*α* function by blocking its ability to activate its receptors on cells in vivo. Leptin has strong metabolic effects, but so do resistin and adiponectin [74]. Therefore, although we do not completely understand the meaning of the elevation of any particular cytokine, the results do show that the stress induced by exercise over the immune system can be modulated by physical training. Another important aspect from these findings and that is hard to be discussed is that, in a fatigue point at the AT intensity, the inflammatory response appears to be the same. This finding suggests that, independent of the physical fitness level of a given individual, the inflammatory response to maximal effort is the same, revealing an interesting setpoint of regulation of immune responses.
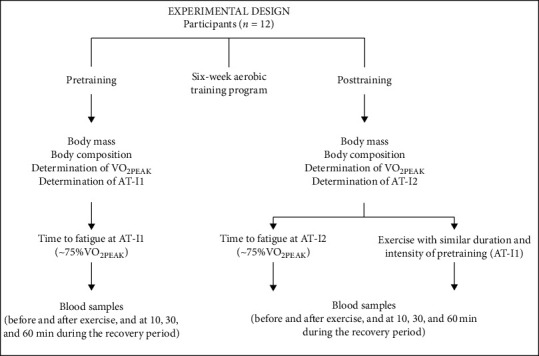


At this stage, it is not simple to correlate the relevance of the training of cytokine responses to any beneficial effects of exercise on health or disease. It is possible that training of cytokine responses may favor the ability of humans to deal better with chronic inflammatory and metabolic diseases, because it tunes down—but does not completely inhibit—the intensity of inflammation. A similar beneficial effect may occur in the context of infection. Indeed, inflammatory responses are necessary for infection but may cause tissue damage if excessive [70]. By training the immune system and fine-tuning cytokines, chronic regular exercise may modulate the function of the immune system in a beneficial manner. This is an interesting concept because it suggests that the immune system performs a much more physiological function (still to be defined) than before. Indeed, the concept of the immune system was developed from the concept of fighting infection; i.e., the immune system has been defined much more by its pathophysiological than physiological functions [73, 75]. The fact that there are significant changes of immunological responses to exercise suggests that the immune system may play a much more important physiological role than previously understood [75]. This possibility is compounded by findings that immunological responses are modulated by diet and by the microbiota in a normal physiological manner; i.e., the function of the immune system is modulated under normal physiology without the need for disease [75]. Much more knowledge is required to define the exact physiology of the immune system and how it interacts with other physiological systems.

A major limitation of the current study, and indeed of any study that evaluates particular exercise or training protocols, is that the findings may be specific to the training protocol, acute exercise, or age of participants under investigation. We studied inflammatory responses to exercise at the fatigue point at AT intensity, in an attempt to normalize exercise load before and after training. However, our studies do show a physiological effect of exercise on the immune system and suggest that understand the physiology of the immune system may be useful to understand its relevance to disease.

There are many speculations and expectations regarding the benefits of chronic physical exercise to health and particularly to the immune system. In conclusion, our results show that moderate chronic aerobic exercise causes significant “training” for certain cytokine responses, including IL-6, sTNFR1, BDNF, and leptin. Trained immunity is known to be relevant in the context of infection but may contribute to the pathogenesis of certain conditions, such as sepsis [44]. Whether the ability of exercise to train immune responses is relevant in the context of health and disease is not yet known but clearly deserves further investigation.

## Figures and Tables

**Table 1:** The progression of six-week aerobic training program.

Week	Frequency (days·week^−1^)	Time (min)
1	3	24
2	3	27
3	3	30
4	3	33
5	3	36
6	3	39

**Table 2:** Data regardingAbsolute exercise intensity (POT), blood lactate concentration (LAC), heart rate (HR), rate of perceived exertion (RPE), percentage of maximal oxygen uptake (% VO_2PEAK_), and maximal power (% POTMAX).

Variables	Pretraining at I1	Posttraining at I1	Posttraining at I2
Absolute potency POT (W)	150 ± 8	150 ± 8	171 ± 8^&#^
Relative potency % POTMAX	69 ± 3	59 ± 2^&^	68 ± 2^#^
Relative intensity % VO_2PEAK_	73.2 + 3.6	66.1 ± 3.0^&^	71.9 ± 1.6
Duration of exercise (min)	71.9 ± 7.5	71.9 ± 7.5	65.4 ± 7.0
Lactate (mM)	6.4 ± 0.5	4.1 ± 0.3^&^	6.4 ± 0.4
RPE	15	11^&^	14^#^
HR (bpm)	158 ± 4	147 ± 3^&^	156 ± 3^#^

^&^ *p* < 0.05, significantly different from pretraining at I1; ^#^*p* < 0.05, significantly different from posttraining at I1. *n* = 10 (% VO_2PEAK_ at posttraining I2).

## Data Availability

Data cannot be shared regarding this paper.
